# Cost analysis of single-use versus reusable bronchoscopes in German intensive care units

**DOI:** 10.3205/dgkh000649

**Published:** 2026-05-15

**Authors:** Nora Leder, Dragiša Mitić, Maria Gundrum, Björn Nowak, Frank Kipp, Sabine Trommer

**Affiliations:** 1 Institute for Infectious Diseases and Infection Control, Jena University Hospital, Jena, Germany; 2 Procurement and Materials Supply Department, Jena University Hospital, Jena, Germany

**Keywords:** intensive care units, bronchoscopy, cost-benefit analysis, infection control, medical device reprocessing

## Abstract

**Aim:**

Bedside bronchoscopy is widely used in intensive care units (ICUs) for a variety of diagnostic and therapeutic procedures. Single-use flexible bronchoscopes (SFB) have emerged as an increasingly utilized alternative to reusable flexible bronchoscopes (RFB), eliminating costs related to device repairs, reprocessing, and routine microbiological surveillance. Beyond clinical outcome parameters, economic and organizational factors as well as aspects of patient safety, including the availability of diagnostic procedures and infection prevention considerations, play an important role in the choice between RSB and SFB.

**Methods:**

Using a cost-minimization approach, we compared the costs associated with RFB and SFB during a two-year study period in a German tertiary care hospital with two surgical and two non-surgical ICUs, identifying key cost drivers, assessed break-even points under different utilization scenarios through sensitivity analyses, and evaluated their implications for procurement decisions and implementation strategies in the ICU setting.

**Results:**

During the study period from 1 January 2023 to 31 December 2024, on average 1,253 bronchoscopic procedures were performed in the ICU setting. Cost per procedure were € 346.19 per use for SFB and € 97.28 for RFB with a break-even point for cost effectiveness of RFB of 317 procedures per year. Sensitivity analysis showed robustness of the results in our setting of high procedure volume to variable maintenance costs and reduction in investment costs of SFB.

**Conclusion:**

For the German ICU setting with in-house reprocessing and high procedure volume, RFB is cost effective compared to SFB.

## Zusammenfassung

**Zielsetzung:**

Bettseitige Bronchoskopien sind unverzichtbarer Bestandteil des Atemwegsmanagements auf Intensivstationen. Der Einsatz von Einwegbronchoskopen (SFB) stellt eine Alternative zu wiederverwendbaren Bronchoskopen (RFB) dar und vermeidet Kosten für Reparaturen, Aufbereitung und mikrobiologische Überwachung. Neben klinischen Endpunkten sind ökonomische, organisatorische und infektionspräventive Aspekte entscheidend für die Entscheidung zwischen SFB und RFB.

**Methoden:**

In einer Kostenminimierungsanalyse wurden die Kosten von RFB und SFB über einen zweijährigen Zeitraum vom 01.01.2023 bis 31.12.2024 in einem deutschen Krankenhaus der Maximalversorgung mit vier Intensivstationen verglichen. Es wurden Kostentreiber identifiziert, Break-even-Punkte berechnet und Sensitivitätsanalysen für unterschiedliche Nutzungsszenarien durchgeführt, um Beschaffungs- und Anwendungskonzepte zu bewerten.

**Ergebnisse:**

Im Mittel wurden 1.253 Bronchoskopien pro Jahr durchgeführt. Die Kosten pro Anwendung betrugen 346,19 € für SFB und 97,28 € für RFB. Der Break-even-Punkt lag bei 317 Anwendungen pro Jahr. Die Sensitivitätsanalysen zeigten die Robustheit der Ergebnisse gegenüber steigenden Wartungskosten sowie gegenüber sinkenden Anschaffungskosten für SFB.

**Fazit:**

In einem Hochvolumen-Intensivstationssetting mit In-house-Aufbereitung sind RFB gegenüber SFB kosteneffektiv.

## Introduction

Bedside bronchoscopy for diagnostic and therapeutic procedures in intensive care units (ICUs) poses procedural challenges, because commonly used reusable flexible bronchoscopes (RFB) cause substantial logistical and economic demands, including high investment costs, reprocessing, repairs, microbiological surveillance, and complex process logistics [1].These processes require additional departments outside the ICU, which have to be available 24/7 to ensure constant availability of RFB in the ICU.

Single-use flexible bronchoscopes (SFB) provide comparable image quality and procedural outcomes compared to RFB, while also being perceived as easier to use by operators [2] and are an alternative, particularly in situations when reprocessing is not available, in patients with prion diseases where reprocessing options are limited [3] or in acute emergency scenarios requiring immediate device availability. Beyond clinical outcome parameters, economic and organizational factors as well as aspects of patient safety, including the availability of diagnostic procedures and infection prevention and control (IPC) considerations, play an important role in the choice between RSB and SFB [1].

Previous cost analyses conducted in European and international healthcare settings have demonstrated that the cost-effectiveness of reusable bronchoscopes is strongly dependent on procedure volume [1]. However, no studies to date have evaluated the economic implications of RFB versus SFB specifically in German ICU settings. 

Using a cost-minimization approach, we compared the costs associated with RFB and SFB in a German ICU setting, identifying key cost drivers, assessed break-even points under different utilization scenarios through sensitivity analyses, and evaluated their implications for procurement decisions and implementation strategies in the ICU setting.

## Methods

We conducted the cost-effectiveness analysis based on the CHEERS-Checklist for reporting of economic evaluations of health interventions [4].

This study was conducted at the four ICUs in our 1,400 beds tertiary care university hospital from 01.01.2023 to 31.12.2024. The two surgical ICUs (ICU I and II) comprise of 20 beds each and the two non-surgical ICUs with ten (ICU III) and 14 (ICU IV) beds, respectively. Our in-house central sterile services department (CSSD) provides 24/7 endoscope reprocessing services following the nationally recommended reprocessing workflow [3].

RFBs are stored in the ICU and used for bedside bronchoscopy. After use, the devices are transported by the internal hospital transport service to the CSSD for reprocessing. Following reprocessing, the bronchoscopes are returned to the ICU via the same transport service.

All bronchoscopic procedures performed during the study period were included. Parameters used for cost calculations were total number of bronchoscopic procedures performed, number of available reusable bronchoscopes, investment costs, maintenance and reprocessing costs associated for RFB and SFB use. Data on turn-around-time for RFB reprocessing was not systematically available.

Clinical health outcomes were not assessed in this analysis.

### Measurement of resources and costs

A single study-based estimates approach was used for the economic evaluation. Data were obtained from institutional records, manufacturer information, and empirically measured workflow parameters. Annual depreciation was calculated based on the expected service life defined in the German depreciation tables for the healthcare sector published by the Federal Ministry of Finance [5].

Hourly wages were derived from the collective wage agreement for public sector employees of the German federal states (TV-L) and the collective wage agreement for physicians at German university hospitals (TV-Ä). Monthly gross salaries including the university allowance were used to calculate hourly personnel costs.

Costs per procedure for SFB included variable acquisition costs of SFB per procedure and fixed deprecation costs for light sources. Disposal costs of SFB were not included.

The average cost per procedure for RFB was calculated using a cost model including fixed and variable cost components. Annual fixed costs comprised costs for: depreciation (RFB, washer-disinfectors for endoscopes (RDG-E), bronchoscopy towers, light sources), repair, maintenance, validation, personal (IPC and medical Microbiology specialists) and consumable costs for microbiological surveillance. Costs associated with drying cabinets were not included, as these devices are not used for RFBs the ICU setting.

Variable costs consisted of consumable material and personal costs for reprocessing per procedure. Costs for consumable material were calculated based on manufacturer-reported prices. Personnel costs were calculated for ICU nursing staff, CSSD personnel, and medical laboratory technicians. 

The duration of the reprocessing workflow was empirically assessed by the IPC team. Costs related to water consumption (softened and demineralized water), wastewater, electricity, and compressed air were not included in the reprocessing cost calculations, as the proportion attributable to reprocessing of bronchoscopes used in the ICU is difficult to allocate and considered negligible relative to total costs. 

### Sensitivity analysis

A univariate sensitivity analysis was conducted assuming all bronchoscopies during the study period were either performed with only RFB or SFB. The break-even point of procedures per year *(N)* considering fixed costs *(F)* and variable costs *(v)* for RSB and SFB was calculated as:


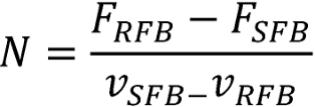


Modeling scenarios included variation in annual procedure volume (200–1,500 procedures), a 50% increase in repair, maintenance, and validation costs for reusable bronchoscopes, and a 20% variation in acquisition costs for single-use bronchoscopes. These ranges were selected based on observed price developments in recent years and are intended to reflect plausible cost increases and reductions associated with discount agreements, market dynamics, and pricing variability.

All costs are reported in Euro (€). Costs are presented as gross prices, including 19% value-added tax (VAT) without discounting.

## Results

During the study period from 1 January 2023 to 31 December 2024, on average 1,253 bronchoscopies were performed in ICU I-IV. A total of 20 reusable bronchoscopes (16 flexible fiber endoscopes Karl Storz^®^, 4 flexible bronchoscopes Olympus^®^) were available (Table 1 (Tab. 1)). Informal feedback on rationale behind RFB or SFB use revealed heterogeneous decision-making criteria, often driven by users’ subjective risk perception. 

During the study period, Ambu^®^ aScope 4 and Ambu^®^ aScope 5 were available as SFB with mean investment costs of € 344.15 per SFB and € 2,558.50 annual deprecation costs for the available one light source per ward. The total annual fixed costs for RFB during the study period were € 108,258.02 (Table 2 (Tab. 2)).

In the sensitivity analysis, the break-even point, at which reusable bronchoscopes become less costly than single-use bronchoscopes, was calculated as 317 procedures per year.

In the base-case scenario of the study hospital with 1,253 procedures per year, the cost per RFB was 97.28 €, corresponding to a cost that is more than 3-fold lower than that of the SFB alternative (346.19 € per procedure) (Table 3 (Tab. 3)).

In the sensitivity analysis, increasing repair, maintenance and validation cost of 50% in the base-case scenario (1,253 procedures with RFB per year), resulted in


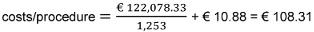


per procedure for RFB and a break-even-point of 358 procedures per year.

Additionally, a 20% price reduction for SFB (e.g., due to discount agreements or market competition) was simulated. Under these assumptions cost in the base-case scenario for SFB was € 277.36 per procedure and a break-even point of 399 procedures. 

## Discussion

Here, we present to our knowledge the most comprehensive cost-effectiveness analysis regarding the use of SFB and RFB for the German in-patient sector from an economic and IPC perspective.

Our analysis demonstrates the cost-effectiveness of RFB in a German ICU setting with high bronchoscopy frequency, in-house reprocessing and IPC-quality control. The main variables influencing the break-even point in our analysis were number of procedures performed. Only in scenarios with drastic reduction of annual procedure numbers (<200 bronchoscopies), SFB would be cost effective, which aligns with international studies, who report an average break-even point of 306 annual procedures [1]. Despite modeling for substantially higher repair, maintenance, and validation costs, reusable bronchoscopes remained considerably less costly than single-use devices, indicating that the results are robust to variations in these cost components. So, for our real-world scenario of frequent bronchoscopies in the ICU, higher fixed costs for RFB due to maintenance or lower investment costs for SFB did not favor SFB use. 

The main cost drivers were investment costs. Personal and material cost for reprocessing had only a small percentage in the total costs for RFB and can be neglected, especially in scenarios like the described where the infrastructure of in house CSSD and IPC are established regardless of bronchoscopy use in the ICU. Besides costs, the CSSD infrastructure influences the workflow and availability of RFB, thus making it a relevant factor for determining the number of bronchoscopes needed. Data on turn-around-time for RFB reprocessing was not systematically available in our study, which hindered integration of this factor in our analysis. 

Considering the Diagnosis-Related Group (DRG)-based German reimbursement system, a precise knowledge of the metrics of performed procedures and in house reprocessing turn-around-time are key for informed and economic optimized decision making.

From an IPC-perspective, there are no strict indications for the use of SFB other than the use in patients with suspected prion disease or as contingency strategy, if reprocessing is not available [3]. Since the incidence for prion disease in Germany is low [6], only a limited number of SFB might be sufficient. Tools like bronchomix [7] can help to estimate the number of RFB and SFB needed for a hybrid use model. The rationale for the use of RFB and SFB was discussed with doctors performing bronchoscopy in the ICU setting and the results of this analysis will be incorporated in future investment strategies for SFB and RFB in the ICU setting of our hospital.

### Limitations

We did not include costs for water, electricity or disposal costs, because we considered them no economically relevant, however, they are relevant from a sustainability point of view. Despite these resources being used in reprocessing, life cycle assessments show lower greenhouse gas emissions (GHG) of RFB compared to SFB [8]. Another study estimated a break-even point for GHG in favor of RFB after 50 procedures [9], which further supports our analysis in favor of RFB. Due to the high dependency of the data to the number of RFB available and metrics and availably of reprocessing, the generalizability of the calculated break-even points are limited. Further studies should also focus on alternative investment strategies like pay per use- or leasing-models.

## Conclusions

For the German ICU setting with in-house reprocessing and high procedure volume, RFB is cost effective compared to SFB. This investigation highlights the role of IPC as a link between clinical and operational domains, acting as a mediator to support both clinical practice and economic decision-making.

## Notes

### Competing interests

The authors declare that they have no competing interests.

### Funding

None. 

### Authors’ ORCIDs

- Leder N: https://orcid.org/0009-0005-1465-1853
- Mitic D: https://orcid.org/0009-0009-0977-1067
- Kipp F: https://orcid.org/0009-0006-3360-489X
- Trommer S: https://orcid.org/0009-0007-2482-1398

## Figures and Tables

**Table 1:**
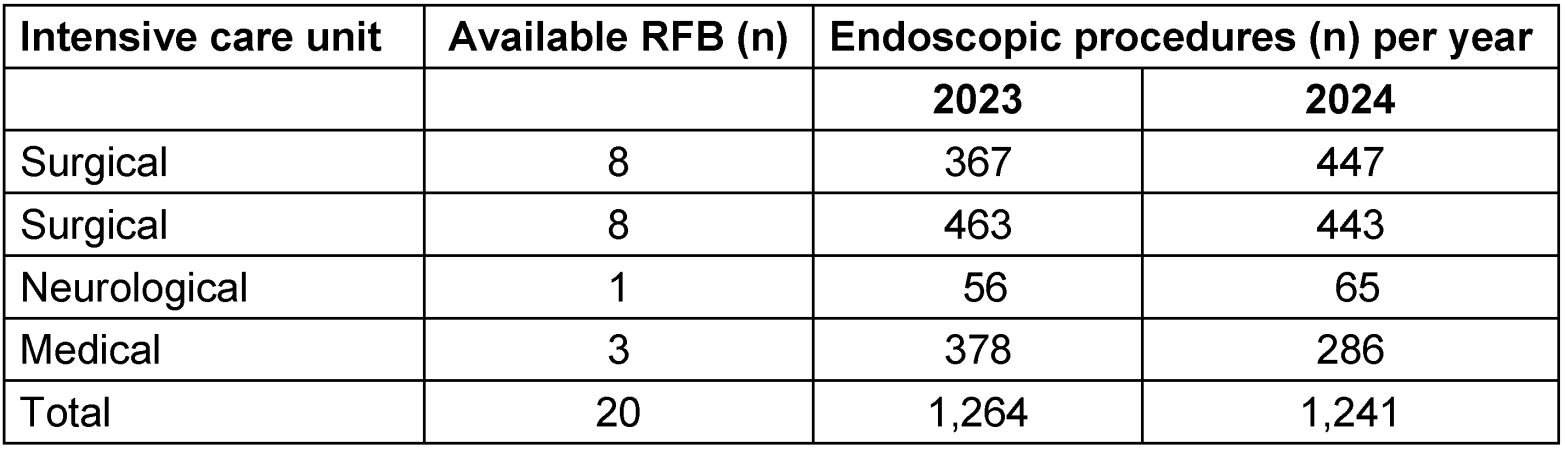
Number of endoscopies performed using reusable endoscopes (RFB) during the study period (2023–2024)

**Table 2:**
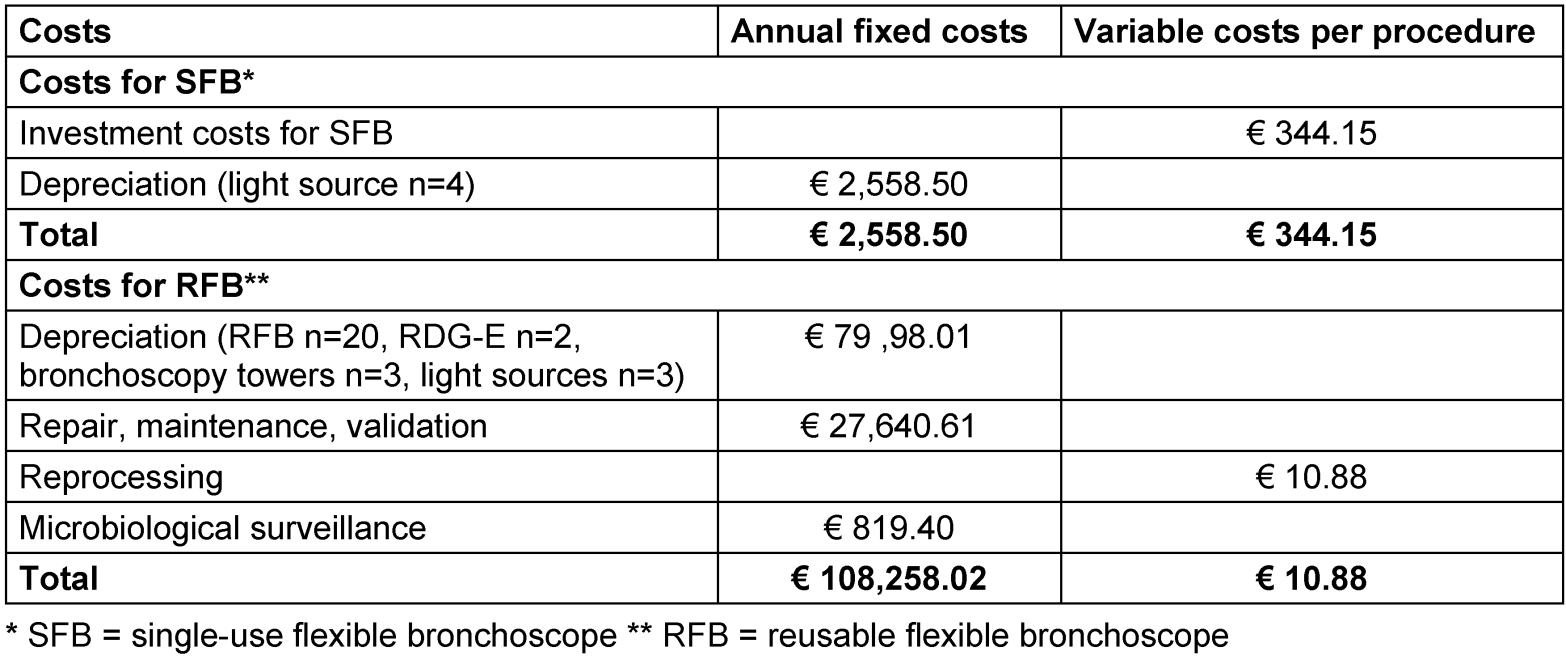
Annual fixed and variable costs per procedure for SFB and RFB

**Table 3:**
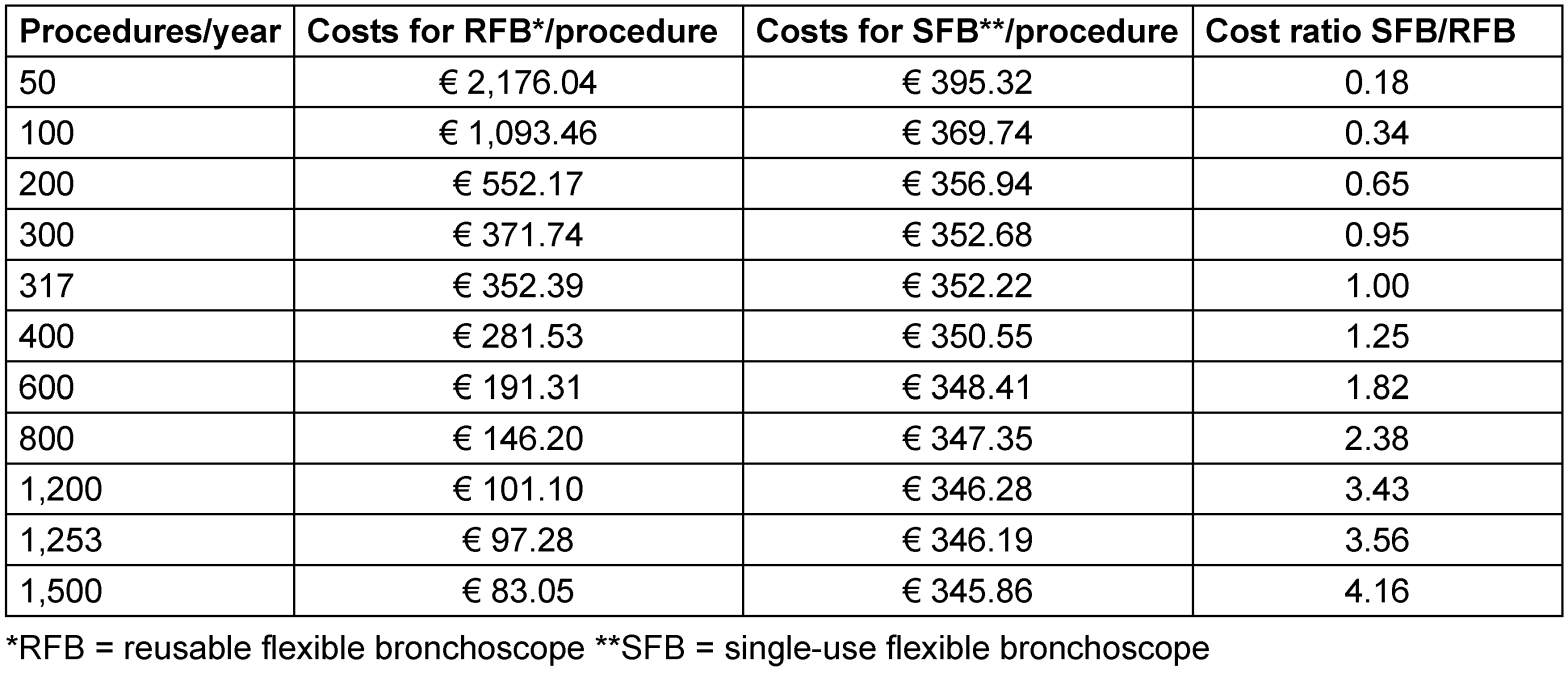
Cost comparison of RFB and SFB across different annual procedure volumes
